# Significant Association Between Adiponutrin and Hepatocellular Carcinoma Risk

**DOI:** 10.1097/MD.0000000000002019

**Published:** 2015-10-30

**Authors:** Hong-Guang Li, Fang-Feng Liu, Hua-Qiang Zhu, Xu Zhou, Jun Lu, Hong Chang, Jin-Hua Hu

**Affiliations:** From the Department of Hepatobiliary Surgery (H-GL, F-FL, H-QZ, XZ, JL, HC) and Department of Gastroenterology Surgery (J-HH), Shandong Provincial Hospital Affiliated to Shandong University, Jinan, Shandong, China.

## Abstract

*ADPN* I148M polymorphism has been consistently reported to play a role in liver-associated diseases, such as alcoholic liver disease, chronic hepatitis C, and liver fat and fibrosis in nonalcoholic fatty liver disease. This significant association was also indicated in a series of hepatocellular carcinoma (HCC) studies, where the significance may be affected due to the small sample sizes. The aim of this study was to reexamine the *ADPN*-HCC association by use of meta-analysis. Biweekly computer-based literature searches plus manual screening were undertaken in an effort to identify all studies that met the predefined inclusion criteria. The Mantel–Haenszel method was selected to estimate risk effects (odds ratio [OR] and 95% confidence interval [CI]). To examine reliability of the pooled risk effects, we additionally performed sensitivity analysis and publication bias tests. Ten studies (1335 HCC patients and 2927 HCC-free controls) were identified for the meta-analysis. We found significantly increased risk of HCC attributable to presence of *ADPN* I148M polymorphism, with the highest risk associated with the M/M genotype under the recessive model of inheritance (OR = 2.23, 95% CI = 1.87–2.67, between-study heterogeneity: *P* = 0.468). The significant increase persisted in Caucasian and African when data were stratified by ethnicity. Subgroup analysis according to source of controls revealed similar risk effects. Our meta-analysis indicates that I148M polymorphism in the *ADPN* gene may independently contribute to the progression of HCC irrespective of the etiologies.

## INTRODUCTION

Hepatocellular carcinoma (HCC), a known multi-etiologic malignancy in nature, represents the most common histological subtype of primary liver cancer and is currently being thought of as a growing health problem across the global.^1,2^ Viral-related cases are overwhelmingly associated with persistent infections by hepatitis B virus (HBV) and hepatitis C virus (HCV), and most nonviral-related cases could be attributable to tobacco smoking, excessive alcohol consumption, overweight, diabetes, liver steatosis, and familial/genetic factors (eg, absence of alpha-1-antitrypsin, inherited hemochromatosis).^3–7^ The identification of genetic inheritance as an important risk factor for HCC^8,9^ leads to the hypothesis that single-nucleotide polymorphisms (SNPs) in candidate genes may act as susceptibility factors for the prevalent liver disease.

Adiponutrin (*ADPN*) corresponding to patatin-like phospholipase domain-containing protein 3 (*PNPLA3*) is a multifunctional enzyme encoded by the human *ADPN* gene at long arm of chromosome 22 at band 13.31.^10^ The single-pass type II membrane protein is a triacylglycerol lipase involved in the mediation of triacylglycerol hydrolysis in adipocytes. The same hormonal pathways regulating fat metabolism in the liver function as mediators of *ADPN* activities.^11,12^ A common nonsynonymous SNP (I148M) located at exon 3 of *ADPN* with a C to G substitution has been consistently associated with liver-related diseases, such as alcoholic liver disease, chronic hepatitis C, and liver fat and fibrosis in nonalcoholic fatty liver disease.^13–15^

Nevertheless, its role in HCC occurrence remains poorly understood. A variety of groups have focused their research on the association of I148M polymorphism with HCC susceptibility, failing to provide convincing evidence for the *ADPN*-HCC association most likely because of sample insufficiency of their published studies. For example, Valenti et al^16^ analyzed a total of 325 cirrhosis patients with (n = 50) or without HCC (n = 275), demonstrating evidence of 2.16 times increased risk in relation to the M/M genotype of I148M polymorphism.

In order to better define the pathological role of I148M polymorphism in HCC occurrence, we decided to perform a meta-analysis of all epidemiological data in agreement with the statement of PRISMA (preferred reporting items for systematic reviews and meta-analyses).^17^

## METHODS

### Literature Search Strategy

Literature searches were undertaken through Cochrane Central Register of Controlled Trials, ISI Web of Science, Wiley Online Library, PubMed, and Embase databases to cover all possibly relevant articles. The keywords were comprised of liver diseases, HCC, *ADPN*, *PNPLA3*, polymorphism, polymorphisms, and variants. We imposed no limits on language and the minimal sample size. To identify the publications that may be missed during computer-based searches, we manually screened the reference lists of review articles and the original articles with available data on *ADPN* I148M polymorphism and HCC occurrence. The research was approved by the ethical committee of Shandong Provincial Hospital affiliated to Shandong University. Written consents were obtained from all patients.

### Inclusion Criteria

The criteria including the case group consisted of histologically confirmed HCC patients, the controls must be cancer-free subjects, the genetic contribution of *ADPN* I148M polymorphism to HCC risk must be investigated, and no departure from Hardy–Weinberg equilibrium (HWE) in controls, were designed for the studies that could be included in the meta-analysis. We did not consider the studies violating any item listed above.

If the same patient population was used in several publications by the same authors, we selected the most informative publication containing the largest number of individuals.

### Data Extraction

Based on a standard protocol and data-collection form made according to the inclusion criteria, 2 investigators separately extracted first author’ last name, publication date, total cases and controls, study country, ethnicity, study design (case–control or cohort), source of controls, minor allele frequency (MAF) of controls whenever accessible, source of DNA, mean age, and count of cases and controls with *ADPN* I148M polymorphism genotypes. Conflicting evaluations were settled through discussion with the most senior investigator of this study.

### Statistical Analysis

We first checked the HWE deviation using χ^2^ test among control populations to guarantee that all studies included in the meta-analysis were in HWE. Pooled odds ratios (ORs) with 95% confidence intervals (CIs) were computed to estimate the effects of *ADPN* I148M polymorphism on HCC. The statistical significance was determined with the Z-test. Pooled ORs were obtained by assuming the allele model of inheritance, the homozygous model of inheritance, the heterozygous model of inheritance, the dominant model of inheritance, and the recessive model of inheritance.

Either the random-effects model or the fixed-effects model derived from the DerSimonian–Laird method and the Mantel–Haenszel method, respectively,^18,19^ was performed to evaluate risk effects of the included studies. Between-study heterogeneity was assessed with χ^2^-based Q test, and statistical significance level was set at *P* < 0.05.^20^ In addition, we used the I^2^ metric to quantify the variance across studies (heterogeneity),^21^ and I^2^ > 50% indicated large heterogeneity. When *P* < 0.05 or I^2^ > 50%, we used the random-effects model to calculate the pooled ORs and 95% CIs; otherwise, we selected the fixed-effects model. Subgroup analysis was performed by ethnicity and source of controls.

Evaluation of publication bias was carried out using the funnel plot and Egger's linear regression asymmetry test.^22^ Stability of the combined risk effects was examined by performing the leave-one-out sensitivity analysis.

Statistical data were done with Stata v. 12.0 (StataCorp LP, College Station, TX). *P* < 0.05 was considered significant.

## RESULTS

### Characteristics of Studies

**FIGURE 1:**
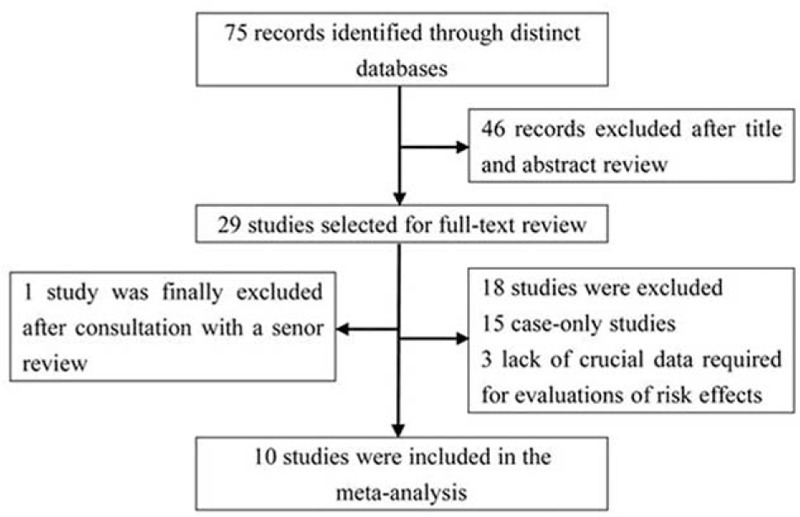
Flow chart showing the detailed selection of studies.

We finally identified 10 studies published over the past 4 years in the meta-analysis,^16,23–31^ after excluding 65 studies for various reasons (systematic reviews, expression-related investigations, reporting on distinct liver diseases except for HCC, absence of the genetic data required for evaluations of risk effects, and case-only investigations), as shown in Figure 1.

Among the eligible studies, most (90%) collected Caucasian as subjects and only 1 study addressed the association in African. Controls in 6 studies were HCC-free cirrhosis patients, in 3 were healthy subjects, and in 1 including both. Six groups isolated DNA genome from blood samples to detect the genotype of *ADPN* I148M polymorphism using different genotyping assays and 4 groups did not report the DNA sources. The mean age in all studies was above 50 years. The quality assessment of all studies was showed in Table 1. The main information is presented in Table 2.

**TABLE 1:**
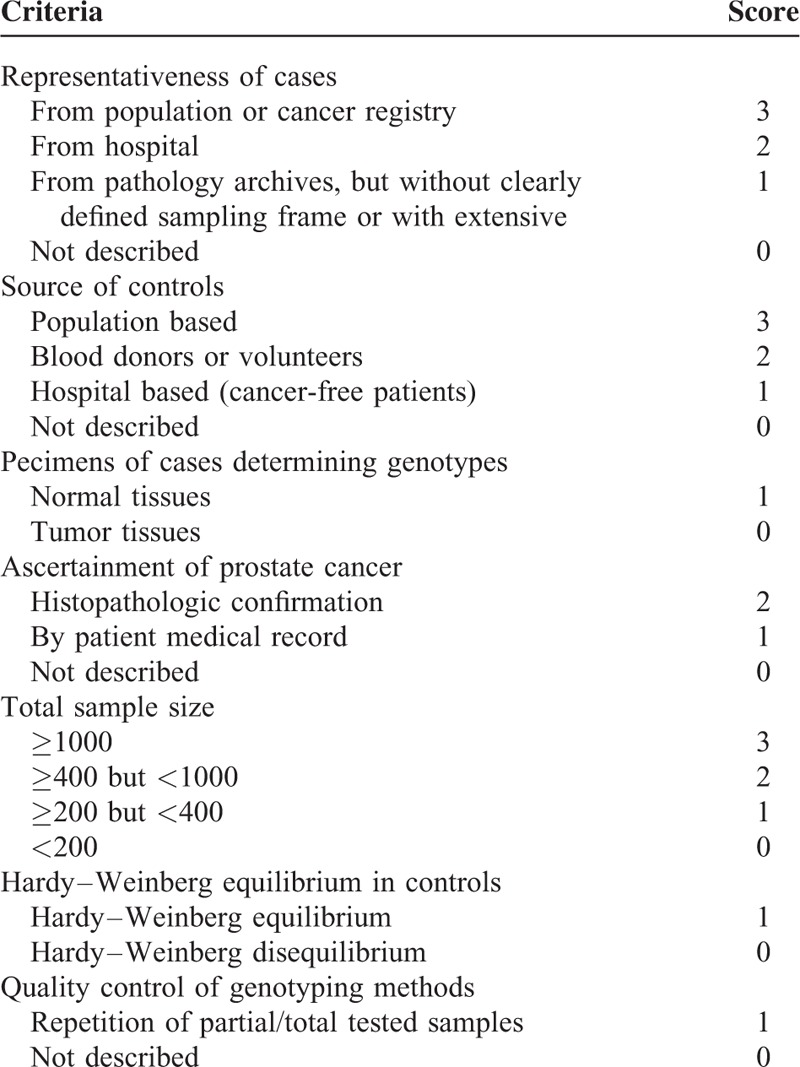
Quality Assessment of Inclusion Publications

**TABLE 2:**
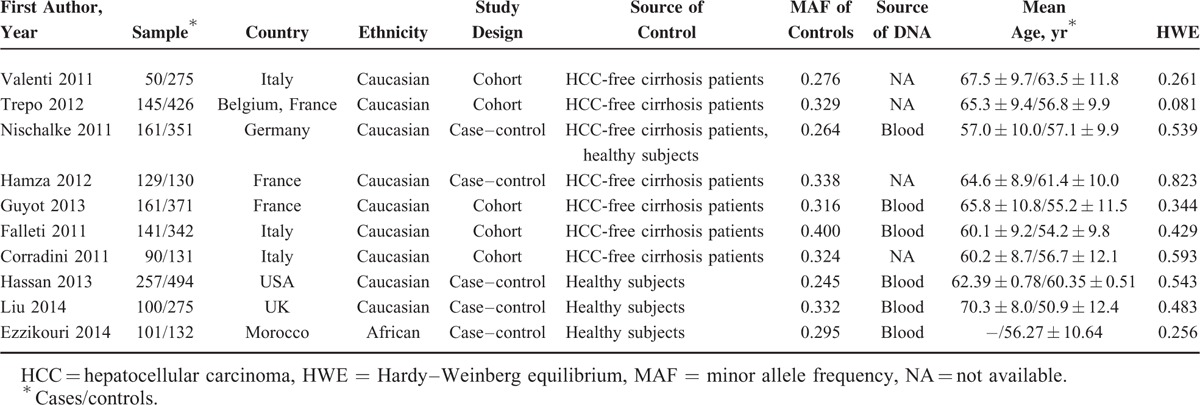
Characteristics Summarized for the Studies Included in the Meta-Analysis

### Main Results

Table 3 lists the primary meta-analysis results derived through overall and subgroup analyses. As there was no indication of significant heterogeneity in Q test, and the I^2^ metric (*P* > 0.05 and I^2^ < 50%, Table 3), the risk effects were estimated by use of the fixed-effects model.

**TABLE 3:**
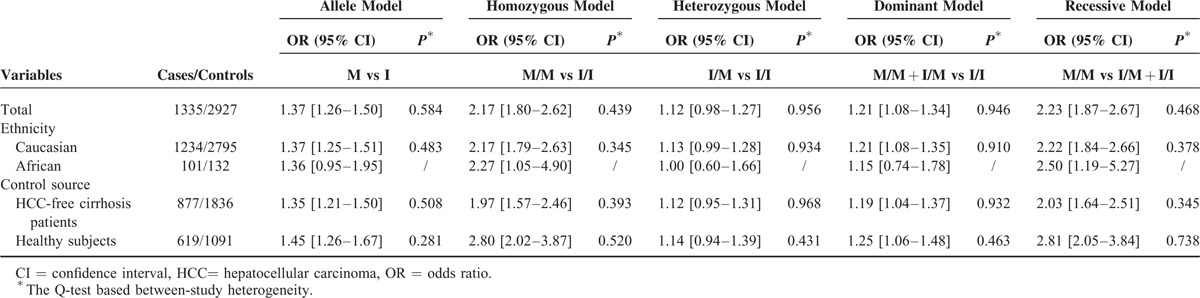
Meta-Analysis of the Association Between ADPN I148M Polymorphism and HCC Risk Under Distinct Genetic Models

### Overall Risk of HCC

**FIGURE 2:**
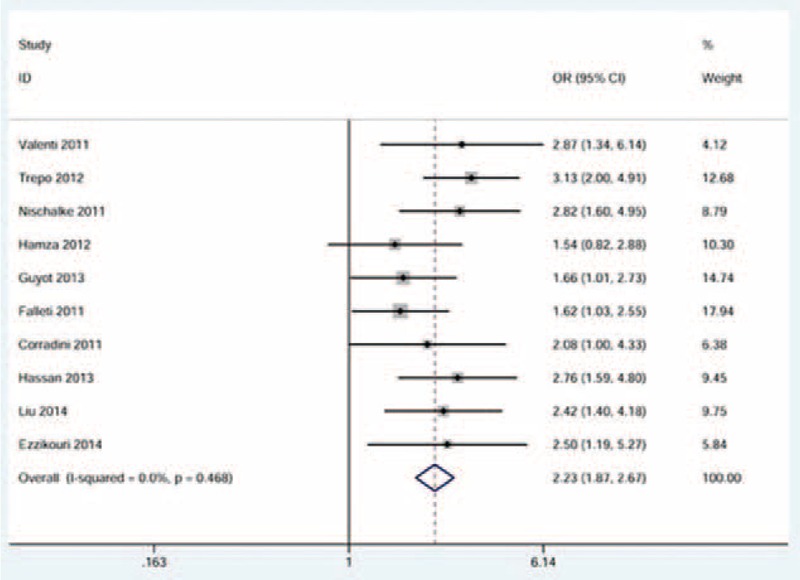
Forest plot for the association between ADPN I148M polymorphism and HCC risk using the recessive model of inheritance. The squares and horizontal lines correspond to the study-specific OR and 95% CI. The area of the squares reflects the weight. The diamond represents the summary OR and 95% CI. CI = confidence interval; HCC= hepatocellular carcinoma; OR = odds ratio.

**FIGURE 3:**
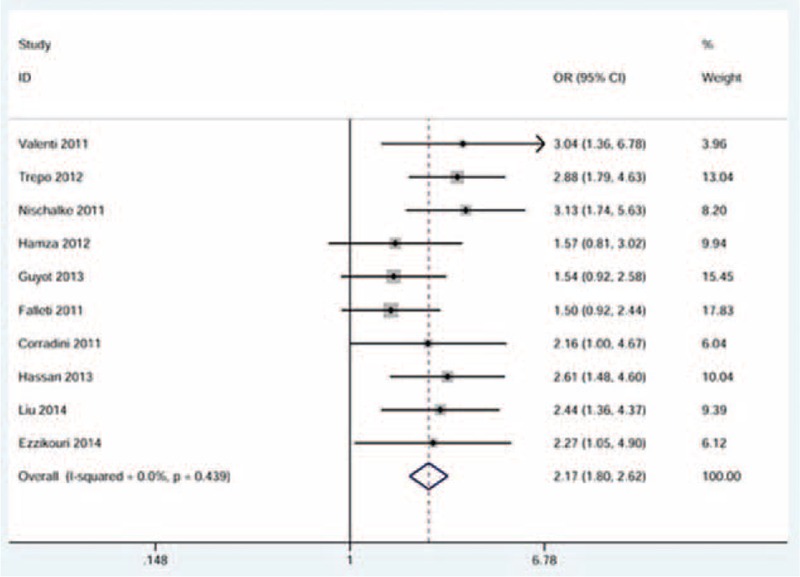
Forest plot for the association between ADPN I148M polymorphism and HCC risk using the homozygous model of inheritance. The squares and horizontal lines correspond to the study-specific OR and 95% CI. The area of the squares reflects the weight. The diamond represents the summary OR and 95% CI. CI = confidence interval; HCC= hepatocellular carcinoma; OR = odds ratio.

When the pooling data set including 1335 cases and 2927 controls was analyzed, we found a strong association between *ADPN* I148M polymorphism and HCC risk. The risk effect was more pronounced in the recessive model of inheritance (OR = 2.23, 95% CI = 1.87–2.67, between-study heterogeneity: *P* = 0.468, Figure 2). Such a high risk was also observed in the homozygous model of inheritance (OR = 2.17, 95% CI = 1.80–2.62, between-study heterogeneity: *P* = 0.439, Figure 3). In addition, a relatively lower risk was shown in the allele model of inheritance and the dominant model of inheritance (Table 3).

### Subgroup Analysis

In the subgroup analysis by ethnicity, we observed significantly increased risk of HCC in all genetic models with the possible exception of the heterozygous model of inheritance in Caucasians (OR = 1.13, 95% CI = 0.99–1.28, between-study heterogeneity: *P* = 0.934, Table 3).

To examine whether source of controls had confounding effects on the genetic association, we stratified the data according control source. The significant increases persisted in the studies with HCC-free cirrhosis patients and those with healthy controls (Table 3).

### Sensitivity Analysis

The influence of individual studies on the pooled risk effects was examined by performing the leave-one-out sensitivity analysis. The ORs and 95% CIs remained stable when any study was excluded, suggesting stability of the results (figure not shown).

### Evaluation of Publication Bias

**FIGURE 4:**
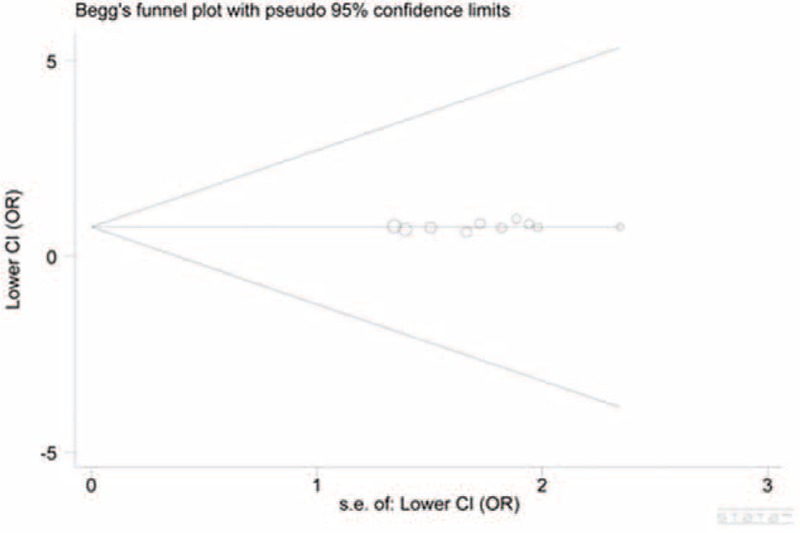
Funnel plot for the heterozygous model of inheritance indicated that no publication bias existed. Each circle corresponds to 1 study (Stata 12.0).

**FIGURE 5:**
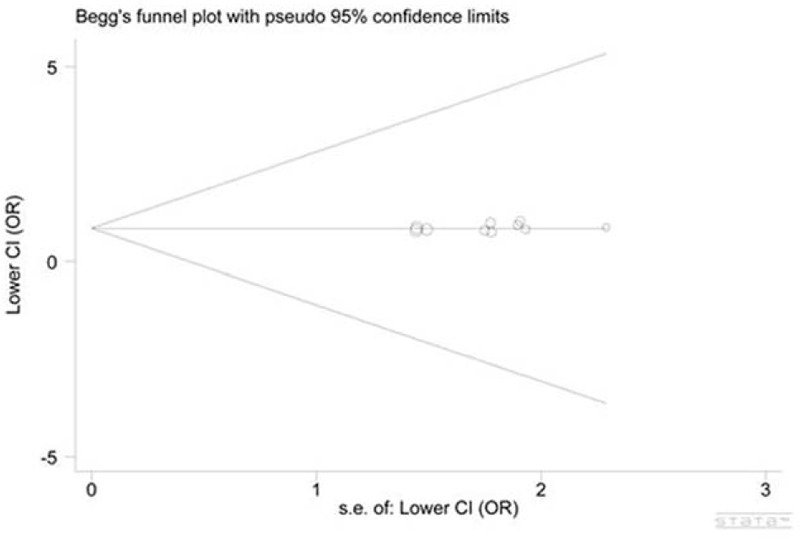
Funnel plot for the dominant model of inheritance indicated that no publication bias existed. Each circle corresponds to 1 study (Stata 12.0).

We constructed the funnel plot for all genetic models, with no one showing obvious asymmetry (Figs. 4 and 5 for the heterozygous model of inheritance and the dominant model of inheritance, respectively). The symmetry was subsequently assessed by Egger's test providing statistical evidence of no funnel plot asymmetry, indicating the absence of publication bias in the present study (*P* = 0.358 and *P* = 0.282, respectively).

## DISCUSSION

Meta-analysis, a statistical method to contrast and combine results from different studies, is widely used to uncover the masked or underestimated SNP-cancer associations reported in single studies.^32^ In view of a less precise measure of interest caused by small samples of the identified studies, we performed a meta-analysis with an aim to achieve a higher statistical power for the association between *ADPN* I148M polymorphism and HCC risk.

A total of 10 studies, providing 1335 cases and 2927 controls, were included in the comprehensive analysis. The derived risk estimates revealed significant genetic contribution of *ADPN* I148M polymorphism to the malignant progression of HCC. We identified that the individuals carrying the rare homozygote (M/M) had more than 2-fold greater risk to develop the malignancy as compared to the individuals with the wide-type homozygote (I/I) alone or in combination with the heterozygote genotype (I/M). Similar risk effects were observed in Caucasians, because only 1 small study investigated subjects of African ancestry. Unexpectedly, not only studies based on HCC-free cirrhosis patients but also those on the basis of healthy subjects showed a significantly increased risk of HCC in association with *ADPN* I148M genotypes. Although these results are obtained based on all published data to date, they remain to be verified in subsequent larger investigations, as we cannot rule out the possibility of false positives due to the currently limited sample.

Our findings are consistent with an earlier meta-analysis examining the effects of *ADPN* I148M genotypes on the development of HCC. In this work, Singal et al demonstrated that PNPLA3 is an independent susceptibility factor for HCC among patients with nonalcoholic steatohepatitis or alcohol-related cirrhosis^33^; the observation derived from the aggregation of 6 studies (including an outlier in which all subjects were HCC patients)^34^ seems to less precise relative to our study, where 4 additional data sets were identified^25,28,29,31^ and 1521 more subjects were analyzed, showing obviously higher risk under the dominant model of inheritance (1.40 vs 2.23). This difference seems to imply that *ADPN* I148M polymorphism is indeed a risk factor for HCC, and only in a study with a sufficient number, can the exact extent of the risk be eventually determined.

HCC has many possible etiologies, such as excessive tobacco use, HBV or HCV infection, heavy alcohol drinking, and liver-related diseases, possibly leading to substantially different susceptibility across the patients with HCC stemming from different carcinogenic agents. Nonetheless, we were unable to consider the etiology of the investigated cancer when examining the association of interest due to data unavailability. This is the first point that needs to be noted in result interpretation.

In addition, risk of HCC, like other cancers, is believed to not be determined by a single agent, but affected by both environmental factors and genetic mutations through complex gene–environment and gene–gene interactions. Thus, the modifying influence resulting from confounding variables should be taken into account to derive a more precise risk effect.

In summary, to the best of our knowledge, this is the largest meta-analysis evaluating the impact of *ADPN* I148M polymorphism on HCC occurrence. The statistical data support that HCC incidence was significantly associated with the nonsynonymous polymorphism. Further studies are warranted to verify our findings and detailed examinations according to HCC etiology and ethnicity are expected to identify the specific at-risk populations.
